# Retrospective review of maternal HIV viral load electronic gatekeeping codes in South Africa

**DOI:** 10.4102/sajhivmed.v25i1.1539

**Published:** 2024-02-20

**Authors:** Siphesihle K. Mahanjana, Tladi Ledibane, Gayle G. Sherman, Tanya Y. Murray, Ahmad F. Haeri Mazanderani

**Affiliations:** 1 Department of Public Health Medicine, Faculty of Health Sciences, School of Medicine, Sefako Makgatho Health Sciences University, Pretoria, South Africa; 2 Centre for HIV and STIs, National Institute for Communicable Diseases, Division of National Health Laboratory Service, Johannesburg, South Africa; 3 Department of Paediatrics and Child Health, School of Clinical Medicine, Faculty of Health Sciences, University of the Witwatersrand, Johannesburg, South Africa

**Keywords:** maternal HIV, electronic gatekeeping codes, viral load, surveillance, vertical transmission prevention

## Abstract

**Background:**

Maternal electronic gatekeeping (eGK) codes for HIV viral load (VL) testing of pregnant and breastfeeding women were developed to permit increased frequency of maternal HIV VL testing without automated gatekeeping cancellation, and to enable virological surveillance.

**Objectives:**

This study describes the national uptake of maternal eGK codes and VL suppression (VLS) rates disaggregated by age during antenatal, delivery and postnatal periods in South Africa during 2022.

**Method:**

HIV VL tests associated with C#PMTCT (used for antenatal and postnatal testing) and C#DELIVERY (used at delivery) eGK codes between 01 January and 31 December 2022, were extracted from the National Institute for Communicable Diseases Data Warehouse. Uptake of eGK codes was calculated using indicators from the District Health Information System as denominators while HIV VLS rates (< 1000 copies/mL) were calculated as monthly and annual percentages.

**Results:**

Overall, national maternal eGK code uptake was 41.8%, 24.5% and 0.12% for the antenatal, delivery and postnatal periods, respectively. The monthly antenatal eGK uptake increased from 27.5% to 58.5% while delivery uptake increased from 17.3% to 30.0%. The overall annual maternal HIV VLS rate was 86.7% antenatally and 87.2% during delivery. The monthly average HIV VLS for adolescent girls and young women (AGYW) was 76.1% antenatally and 79.6% during delivery.

**Conclusion:**

Although overall national uptake of maternal HIV VL eGK codes was low, antenatal and delivery uptake improved over time, thereby facilitating use of eGK codes for programmatic monitoring of maternal VLS rates for the first time. Quality of care among pregnant AGYW requires urgent attention.

**What this study adds:** An automated system for HIV viral load (VL) surveillance of pregnant and breastfeeding women, leveraging the pre-existing National Health Laboratory Service electronic gatekeeping (eGK) code system, which has been in use since 2019. This study highlights the need to strengthen maternal HIV VL eGK use to better monitor the maternal HIV programme.

## Background

Electronic gatekeeping (eGK) is a demand management strategy implemented within South Africa’s National Health Laboratory Service (NHLS) as a cost-saving measure to optimise limited health resources by reducing unnecessary repeat testing.^1,2^ Within the HIV vertical transmission prevention (VTP) programme, inadequate maternal HIV viral load (VL) monitoring during pregnancy and breastfeeding periods poses a significant challenge. Studies report that one in five pregnant women in South Africa lacks VL testing.^3,4^ Maternal eGK codes aim to enhance the national HIV VTP programme in South Africa by permitting more frequent VL testing of pregnant and breastfeeding women by overriding eGK rules. Since 2019, South Africa’s national clinical guidelines have recommended more frequent monitoring of maternal HIV VL compared with the general HIV programme.^5^ Pregnant women on antiretroviral therapy (ART) are expected to have VL testing at their first antenatal care (ANC) visit and ART-naïve clients are recommended to have their first VL test 3 months post ART-initiation during the antenatal period.^5^ Additionally, all pregnant woman are expected to have a VL test at delivery and again 6 months postpartum if virally suppressed.^5^ This aligns with World Health Organization (WHO) guidelines, emphasising maternal HIV viral load suppression (VLS) as crucial for preventing vertical transmission, particularly where emerging evidence suggests that pregnant and breastfeeding women experience frequent episodes of viraemia.^6,7,8^

To ensure maternal HIV VL tests bypass automatic rejection by eGK rules, two novel maternal eGK codes were introduced in 2019: C#PMTCT for HIV VL testing during antenatal and postnatal periods, and a C#DELIVERY code for HIV VL testing at delivery (defined as up to 6 weeks postnatally if no HIV VL was performed at the time of delivery).^5^ According to the guidelines, these eGK codes should be used for all HIV VL requests among pregnant and breastfeeding women up until 6 weeks postpartum.^5^ This is regardless of whether the VL test would have been rejected by gatekeeping cancellation. The rationale for introducing two eGK codes and not three codes (one for each maternal time-point) was to ensure simpler implementation of the codes, thereby supporting uptake. Postnatal testing would be differentiated from antenatal testing by observing whether a patient with a C#PMTCT code had a previous C#DELIVERY code captured within the previous 2 years.

The correct use of the maternal eGK codes also acts as a marker of pregnant and breastfeeding women who undergo virological monitoring within the NHLS. This facilitates the monitoring of the volume of maternal HIV VL testing and suppression during pregnancy, delivery, and breastfeeding periods without additional workload and costs associated with introducing a manually collected national indicator at facility level. Furthermore, the maternal eGK codes have been incorporated within the Results for Action HIV VL reports, which are accessible at a facility and/or district-level and, together with eGK codes, play a valuable role in identifying maternal patients who are virologically unsuppressed, as well as highlighting those with probable virological failure.^9^ This, in turn, supports tracking and tracing teams in the field to recall clients who require urgent clinical follow-up with the aim of improving maternal clinical outcomes as well as working towards eliminating vertical transmission of HIV.^9^

This report describes the uptake of maternal HIV VL eGK codes at both the national and provincial levels, during antenatal, delivery and postnatal periods, between 01 January 2022 and 31 December 2022, and age-disaggregated HIV VLS rates at < 1000 copies per millilitre (copies/mL) and < 50 copies/mL cut-offs.

## Methods

### Study design

A retrospective cross-sectional data review of routine surveillance data was conducted.

### Population

The population comprised all HIV VL tests from South Africa’s public health sector associated with a maternal eGK code registered within the NHLS between January 2022 and December 2022. Women who presented to a private health facility, or who had VLs done that were not associated with an eGK code, were not included.

### Variables

Data were extracted from the National Institute for Communicable Diseases (NICD) Data Warehouse and included HIV VL results, including rejections, age and sex of the patient, and the geographical location (province) of the facility and ward at which sampling occurred. HIV VLS was described as the proportion of tests with an HIV VL < 1000 copies/mL, in keeping with Joint United Nations Programme on HIV/AIDS (UNAIDS) target thresholds, while low-level viraemia was described as the proportion of patients with an HIV VL of 50 copies/mL – 999 copies/mL. As national HIV clinical guidelines define virological suppression as an HIV VL < 50 copies/mL, this cut-off is also reported. The VLS rates were disaggregated by age and described at both the national and provincial levels. The difference between overall maternal HIV VL testing performed and those associated with a maternal eGK code was also assessed by analysing data of the total number of HIV VL tests done (VLD) from wards labelled as antenatal/maternal and delivery/labour/postnatal in comparison to the number of VLs done with an associated eGK during the antenatal and delivery periods, respectively.

### Analysis

**FIGURE 1:**
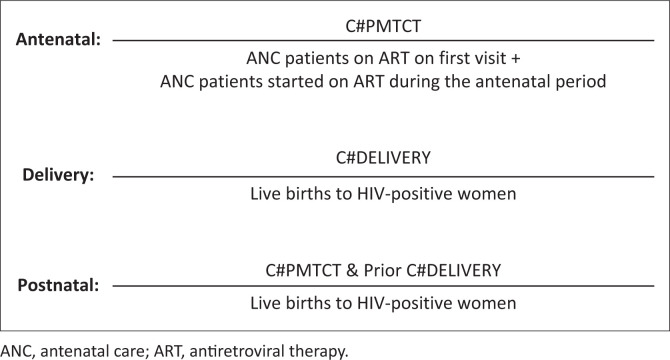
Formulae used to calculate maternal electronic gatekeeping uptake at antenatal, delivery and postnatal time periods between January 2022 and December 2022.

Maternal eGK code uptake during the antenatal period was calculated per month and annually as the number of HIV VL tests with maternal eGK code C#PMTCT as the numerator, excluding patients with a linked C#DELIVERY code within the previous 2 years, and the sum of District Health Information System (DHIS) indicators ‘antenatal patient on antiretroviral treatment (ART) at first visit’ and ‘antenatal patients started on ART during the antenatal period’ as the denominator (referred to hereafter as ‘first ANC visit’). Maternal eGK code uptake during delivery was calculated per month and annually as the number of HIV VL tests with maternal eGK code C#DELIVERY as the numerator and DHIS indicator ‘Live births to HIV-positive women’ as the denominator. The numerator for maternal eGK code uptake during the postnatal period was calculated as the number of HIV VL tests with maternal eGK code C#PMTCT that were associated with a C#DELIVERY code within the previous 2 years. The DHIS indicator ‘Live births to HIV-positive women’ was also used as the denominator. The formulae for the calculations of the eGK uptake during the antenatal, delivery and postnatal periods can be found in Figure 1. The NHLS Corporate Data Warehouse probabilistic record linking algorithm was used to identify patients with a C#PMTCT code who had a prior C#DELIVERY code for categorisation of postnatal versus antenatal testing.^10^ HIV VLS rates were calculated as monthly and annual percentages by using the monthly VLS rates and dividing them by 12 during the antenatal and delivery periods. Furthermore, the VLS rates were disaggregated by age. The eGK codes associated with male gender were calculated by using the numerator as the number of eGK codes associated with male sex and the denominator included the total number of eGK codes during the reviewed period.

## Results

**FIGURE 2:**
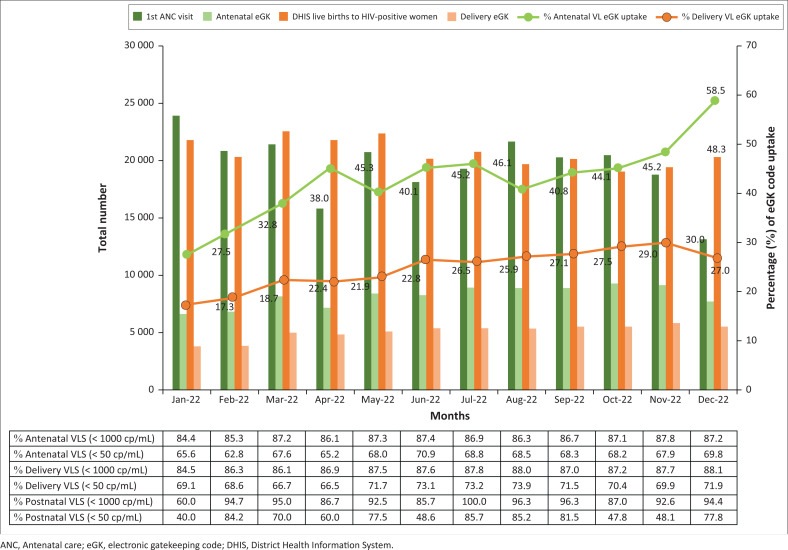
National maternal HIV eGK uptake (antenatal and delivery) and VLS rates between 01 January 2022 and 31 December 2022.

The overall national maternal eGK uptake during the 12-month period was 41.8% (standard deviation [s.d.] = 7.5; *n* = 97 994) for the antenatal period, 24.5% (s.d. = 3.8; *n* = 60 904) for the delivery period and 0.12% (s.d. = 0.0; *n* = 290) for the postnatal period. The monthly maternal eGK uptake ranged from 27.5% to 58.5% for the antenatal period, and 17.3% to 30.0% for the delivery period (Figure 2). Uptake during the postnatal period ranged from 0.05% to 0.18%. The monthly HIV VLS (< 1000 copies/mL) rates remained relatively constant throughout the year for the antenatal (range: 84.4% – 87.7%) and delivery (range: 84.4% – 88.1%) periods (Figure 2). The monthly postnatal VLS (< 1000 copies/mL) rate demonstrated greater fluctuations (range: 60.0% – 100.0%). Overall, during the 12-month period, there was an average monthly HIV VLS (< 1000 copies/mL) of 86.6% (s.d. = 0.9) antenatally; 87.1% (s.d. = 1.0) during delivery; and 90.1% (s.d. = 10.0) postnatally. Furthermore, there were 19.0% (s.d. = 1.5) of patients having low-level viraemia (50 copies/mL – 999 copies/mL) during the antenatal period, 16.5% (s.d. = 1.9) during the delivery period, and 22.9% (s.d. = 11.1) during the postnatal period. The average monthly HIV VLS rate at < 50 copies/mL was 67.6% (s.d. = 2.1) during the antenatal period, 70.5% (s.d. = 2.4) at time of delivery, and 67.2% (s.d. = 16.5) during the postnatal period.

As reported by DHIS, the total number of live births to women living with HIV (WLHIV) between 01 January 2022 and 31 December 2022 was 248 343. During the same period, there were 124 696 (50.2%) HIV VLD in wards categorised as ‘delivery wards’, and the number of C#DELIVERY maternal eGK codes captured was 60 904 (24.5%).

All provinces had higher antenatal maternal eGK code uptake than delivery eGK code uptake between 01 January 2022 and 31 December 2022 (Table 1). The Western Cape province had both the highest antenatal and delivery eGK code percentage uptake of 72.6% and 55.5%, followed by Mpumalanga with 61.4% and 44.4%, respectively. The provinces with the lowest eGK uptake were the Northern Cape, Limpopo, and the Eastern Cape, with the Northern Cape demonstrating negligible uptake at time of delivery of only 3.6%. KwaZulu-Natal had the highest HIV VLS (< 1000 copies/mL) rate of 91.1% during both antenatal and delivery periods, which was considerably higher than the national average of 86.7% (antenatal period) and 87.2% (delivery period). The province with the lowest antenatal suppression rate was the Northern Cape (75.5%) and the lowest delivery suppression rate was Limpopo (80.3%).

**TABLE 1:** Provincial maternal HIV eGK uptake (antenatal and delivery) and VLS rates between 01 January 2022 and 31 December 2022.

Provinces	Antenatal HIV VL eGK uptake (%)	Delivery HIV VL eGK uptake (%)	Antenatal HIV VLS (< 1000 cp/mL) (%)	Delivery HIV VLS (< 1000 cp/mL) (%)
Eastern Cape	24.3	9.2	82.4	85.4
Free State	36.6	26.5	85.9	87.6
Gauteng	58.4	39.8	86.6	85.4
KwaZulu-Natal	31.0	16.3	91.1	91.1
Limpopo	26.8	4.5	81.7	80.3
Mpumalanga	61.3	44.4	86.3	86.9
North West	46.5	14.2	83.3	85.4
Northern Cape	9.8	3.6	75.5	83.8
Western Cape	72.6	55.5	86.9	87.9

**Total**	**41.8**	**24.5**	**86.7**	**87.2**

eGK, electronic gatekeeping code; VL, viral load; VLS, viral load suppression; cp/mL, copies per millilitre.

**FIGURE 3:**
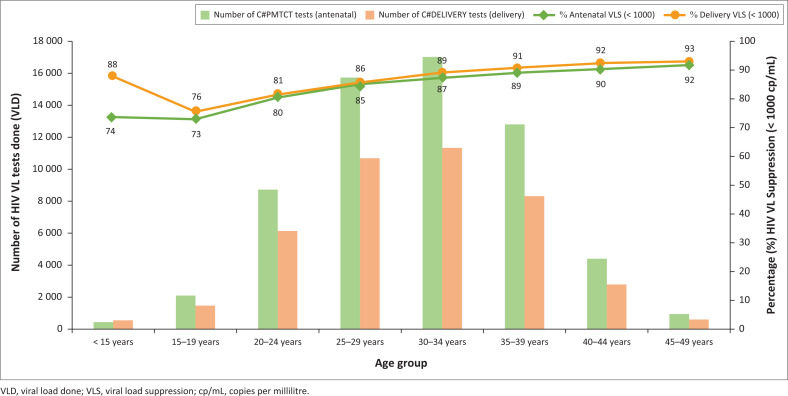
Antenatal and delivery HIV VLS by age group, South Africa, 01 January 2022 to 31 December 2022.

Younger patients generally had lower VLS rates than women belonging to older age groups, with the antenatal and delivery VLS rates ranging from a low of 73.0% and 76.0% in the 15–19-year age group, to a high of 92.0% and 93.0% in the 45–49-year age group, respectively (Figure 3).

There were 769 girls under the age of 15 years with associated antenatal eGK codes, and 818 with associated delivery eGK codes, which constituted 0.8% of all antenatal eGK codes and 1.3% of all delivery eGK codes logged among all age groups (excluding those with an unknown age group). The average monthly HIV VLS (< 1000 copies/mL) rate for girls under the age of 15 years was 74.0% during the antenatal period and 88.0% at the time of delivery.

### Data quality results

A total of 3869 (2.4%) HIV VL results with a maternal eGK code were registered as being from a male patient; 3184 (82.3%) having a C#PMTCT code and 682 (17.6%) having a C#DELIVERY code. Of these, 3058 (79.0%) were taken from either an antenatal (*n* = 1265) or delivery (*n* = 1793) ward. There were an additional 2602 (1.6%) HIV VL tests with a maternal eGK code that were associated with unknown age and 1658 (1.0%) associated with unknown gender.

The total number of HIV VL tests with an associated maternal eGK code that were rejected during the reviewed period was 4096 tests (2.6%). The majority 3022 (73.8%) of these were rejected due to the specimen being unsuitable or insufficient for testing. There were an additional 436 (10.6%) rejected due to eGK rules. Of these, the correct maternal eGK codes were used in 145/436 (33.3%) of cases whereas the remaining 291 (66.7%) were associated with incorrect eGK code usage, including typographical/transcription errors such as #C-LABOUR.

## Discussion

Between January 2022 and December 2022, the overall national uptake of maternal HIV VL eGK codes was low, but there was a marked improvement in the monthly utilisation with antenatal uptake reaching 58.5%, and delivery uptake reaching 27.0%. However, the monthly number of postnatal eGK codes remained negligible. During the 12-month period, the average monthly VLS (< 1000 copies/mL) was 87% during both antenatal and delivery time-points. There was a considerable proportion of patients demonstrating low-level viraemia, with the true VLS (< 50 copies/mL) rate being 68% during the antenatal period and 71% at time of delivery. Because of the low uptake of postnatal codes, it is not possible to confidently report on suppression rates during the breastfeeding period. Provincial variations in eGK code uptake and VLS rates during the antenatal and delivery time-points were evident and require interventions to improve implementation.

### Electronic gatekeeping uptake and implementation gaps

Three implementation gaps contribute to the low uptake of the maternal eGK codes: (1) HIV VL testing not being performed as per clinical guidelines, (2) HIV VL testing done without an associated maternal eGK code on the laboratory test request forms, and (3) maternal eGK codes not being captured correctly within the NHLS.

One potential reason for HIV VL testing not being done as per clinical guidelines is the disruption to the healthcare system caused by the coronavirus infectious disease 2019 pandemic. COVID-19 struck South Africa 3 months after the maternal eGK guidelines were released in December 2019, adversely affecting the training and implementation of maternal eGK codes at both facility and laboratory levels. Thus, healthcare workers may not have fully understood the reasons for introducing the eGK codes or how to use them correctly. The advantage of near real-time monitoring of both HIV VLD and VLS rates during pregnancy, delivery and postpartum, enabled by maternal eGK codes without requiring data capture at facility level, was not appreciated. Furthermore, the pandemic heavily impacted multiple aspects of the HIV programme such as testing and ART initiation, with lockdown restrictions likely having a negative impact on HIV VL monitoring and uptake of the associated maternal eGK codes.^10^ Although the period for which data were analysed coincided with minimal COVID-19 era restrictions, the repercussions of the pandemic on health programmes continues to be experienced.

Another potential factor contributing to the under-utilisation of maternal HIV VL eGK codes by clinicians on laboratory test request forms is the lack of standardised NHLS stationery. As not all NHLS request forms provide a designated space for an eGK code, this further inhibits the uptake of codes. A designated space on test request forms for eGK codes would be useful, not only for maternal patients, but for all patients who may require an eGK code for other reasons. To address this, some districts have opted to use pre-stamped forms. However, the efficacy of this has yet to be reported upon.

Lastly, failure to include the maternal eGK codes on the request forms or appropriately capture the code on the laboratory information system (LIS) by clinical and laboratory staff, respectively, may reflect a lack of understanding of how the maternal eGK codes differ from other eGK codes. Maternal eGK codes need to be logged and captured by the laboratory data capturers, irrespective of whether the specimen would have been rejected by gatekeeping rules, whereas other eGK codes are only captured to override LIS gatekeeping rules that indicate specimen rejection is imminent.

Despite the above challenges, some health districts have successfully improved the uptake of maternal eGK codes by identifying gaps through monthly NICD maternal HIV VL eGK reports detailing code usage at facility level, auditing request forms and targeting specific facility and laboratory staff for problem-solving discussions and/or training.

### Viral load testing during delivery

The results of this study demonstrate that the proportion of HIV VL with a C#DELIVERY eGK code represent only a quarter of all WLHIV at time of delivery, and half of all HIV tests done in ‘delivery wards’. This suggests that HIV VL testing is not being done according to national VTP guidelines. Although not all delivery wards are named, and therefore the number of tests done in ‘delivery wards’ reported here is an underestimate, verification work performed in Tshwane district, where all delivery wards have been mapped in the NICD Data Warehouse, has yielded similar findings (A. Haeri Mazanderani, NICD. Personal communication, 13 April 2023). Hence, there is likely a large proportion of pregnant WLHIV who do not have HIV VL testing performed at time of delivery.^11,12,13^ Furthermore, among WLHIV who have an HIV VLD at the time of delivery, only half have a maternal eGK code correctly captured within the LIS. This discrepancy between VLD and VLD associated with a C#DELIVERY code could be due to maternal eGK codes not being provided on the test request forms and/or not being captured on the LIS. Anecdotal evidence/audits from the field suggest that both of these gaps occur; however, further research is required to determine the extent of these problems across the various health districts.

### HIV viral load suppression

Maternal HIV VL eGK codes readily enable VLS trend analysis, including among subgroups, thereby supporting outcome-monitoring of interventions and programme updates such as the continued roll-out of dolutegravir-based regimens. Importantly, this study demonstrates that the proportion of pregnant clients with a VL < 1000 copies/mL (86.6%) is notably lower than the VLS rate for WLHIV aged 15–49 years, as well as the overall suppression rate within the national ART programme, both reported as 91% during 2022.^14^ The low rates of VLS during pregnancy highlight the importance of enhancing the integration of family planning services with HIV care, including aiming to prevent unplanned pregnancies and improve treatment adherence. This is especially crucial for women in suboptimal health, both for maternal and VTP outcomes, considering infant prophylaxis is determined by maternal delivery HIV VL result. Data from the Saving Mothers Report highlight that inadequate VLS contributes to increased HIV-related deaths among pregnant and postpartum women, where almost 75% of all HIV-related deaths were in women who were not virally suppressed.^15^ Therefore, a greater priority should be placed on sufficient virological monitoring and proactive action by clinicians.^16^ Adolescent girls and young women (AGYW) demand special focus within HIV prevention programmes.^17^ Studies have found that adolescents have lower rates of ART adherence and lower rates of VLS with poorer treatment outcomes compared to both children and adults.^18,19,20^ This study found AGYW to have considerably lower VLS rates compared to older maternal patients. This further emphasises the need to support this especially vulnerable group.^21^ Furthermore, the number of pregnant girls under the age of 15 years is concerning and highlights issues related to health education, underage sex, access to healthcare services and sexual abuse. This is an area requiring more in-depth research as well as appropriate interventions to address and monitor this public health concern.

As the monthly antenatal and delivery suppression rates at national level were fairly constant during the 12-month period, despite notable increases in the uptake of these eGK codes (with percentage coverage nearly doubling), the antenatal and delivery maternal suppression rate findings are likely to be representative of the overall HIV programme. This is in contrast to the monthly postnatal suppression rates, which varied considerably. Improved postnatal VL monitoring and eGK code use is required before these data can be used to represent accurate maternal VLS rates during the breastfeeding period.

### Data quality

The NHLS provides crucial diagnostic pathology information to the South African public health sector, serving over 80% of the population.^22^ These data have been used to monitor numerous diseases and public health programmes including the HIV programme.^22^ Various national surveys have been conducted to evaluate the cost effectiveness of using NHLS data for monitoring the HIV VTP programme.^22^ The accuracy of NHLS data therefore has far-reaching implications and is crucial for ensuring data-driven decision-making.^10^ Nevertheless, with all the measures that have been put in place to ensure accurate data of high quality, data quality challenges were identified. Although they potentially hinder accurate monitoring of the maternal eGK codes, they constitute a small fraction of the total data set. These include maternal eGK codes associated with male sex, those associated with unknown age, as well as those associated with rejected tests including the use of incorrect codes (e.g., C#MATERNITY). The vast majority of the maternal eGK codes associated with male gender were likely to be female patients as nearly 80% of these specimens were taken from wards labelled as ‘antenatal’ or ‘delivery’. However, it is not clear the extent to which the remaining maternal eGK codes registered for male patients reflect transcription/typographical errors or incorrect usage of the codes by healthcare workers. Documentation of incorrect or missing gender is a general problem affecting NICD Data Warehouse test data and generally arises from incomplete or illegible request forms or data capturing errors. Maintaining data quality requires ongoing effort. Furthermore, it is not clear why correctly used maternal eGK codes were rejected for eGK reasons. The majority of specimen rejections were due to sample-related problems, indicating a requirement for further training and quality assurance of phlebotomists and healthcare workers responsible for drawing blood. This is necessary for effective minimisation of rejections.

### Limitations

A number of limitations were identified from the data extracted and analysed. As monthly test-level data were analysed, patients with repeat testing may have reflected more than once, although this is unlikely within any given month. The number of postnatal HIV VLD with an associated maternal eGK code was extremely low. This negatively affects the accuracy of the calculated VLS rates for the postnatal period and, hence, postnatal eGK uptake and VLS rates were not reflected in the figures. As noted within the results, the number of HIV VL with maternal eGK codes are not a true reflection of all HIV VL testing done during pregnancy and breastfeeding on account of implementation challenges associated with eGK usage. Unfortunately, it was not feasible to evaluate the degree to which codes are not provided on request forms compared to those not correctly captured within the LIS by laboratory data capturers. Another limitation is the lack of standardised processes to determine if maternal eGK codes are incorrectly used for non-maternal patients. Challenges also exist with the C#PMTCT code during the postnatal period where a prior C#DELIVERY code is required to be linked in order to be categorised as having postnatal HIV VL. This requires correct utilisation of the C#DELIVERY codes as well as accurate and consistent capturing of patient demographic details within the NHLS to link results. This may have resulted in an inflated number of antenatal codes captured. Furthermore, without a designated postnatal eGK code, incorrect classification of newly diagnosed mothers with HIV VL testing performed postnatally is inevitable. Using DHIS indicators to calculate uptake of maternal HIV VL eGK codes also presents limitations that may have resulted in under-/over-reporting percentage uptake. The indicators have inherent limitations, such as live births to non-singleton pregnancies being counted in the denominator, but not the numerator for calculating uptake of the C#DELIVERY codes.

## Conclusion

The overall national uptake of maternal eGK codes between 01 January 2022 and 31 December 2022 was low, suggesting poor adherence to guidelines for both HIV VL testing and eGK code usage. Nevertheless, the maternal eGK codes have enabled, for the first time, an automated mechanism for reporting on maternal VLS at different stages of pregnancy. The low HIV VLS rates, especially among AGYW, are concerning and increased efforts are required to meet the UNAIDS targets for VLS and elimination of vertical transmission and improve maternal and infant HIV-related outcomes.

### Recommendations

Further research is required to conduct a root cause analysis of the low uptake of eGK codes and the low VLS among AGYW to implement appropriate interventions. It is important to assess the knowledge, attitude, and practices of clinical healthcare workers and laboratory workers to policy changes to address gaps in knowledge. Districts should harness monthly NICD eGK reports to identify gaps and address them in collaboration with their local NHLS laboratories. Furthermore, HIV care training and workshop opportunities should be used to highlight the use and value of the maternal eGK codes to improve utilisation.
